# Intraosseous Lipoma of the Maxillary Sinus: First Documented Case in an Asian Patient and Review of the Literature

**DOI:** 10.1155/crid/6671840

**Published:** 2025-12-10

**Authors:** Eng Seng Yeoh, Tzy Harn Chua, Jacqueline S. G. Hwang, Sathiyamoorthy Selvarajan, Noah B. T. Teo

**Affiliations:** ^1^ Allsmiles Dental Care, Singapore, Singapore; ^2^ Department of Anatomical Pathology, Singapore General Hospital, Singapore, Singapore, sgh.com.sg; ^3^ Oral and Maxillofacial Surgery, Mount Elizabeth Medical Centre, Singapore, Singapore, memc.com.sg

**Keywords:** bone neoplasms, Caldwell-Luc, histopathology, intraosseous lipoma, maxillary sinus, tomography

## Abstract

**Background:**

Intraosseous lipomas are extremely rare benign tumors composed of mature adipocytes, accounting for only 0.1% of all bone tumors. These lesions are particularly uncommon in the maxillofacial region, with only seven cases of intraosseous lipoma in the maxilla reported to date. This report presents the first documented case of an intraosseous lipoma located in the maxillary sinus of an Asian patient.

**Case Presentation:**

A 41‐year‐old female with a history of thalassemia minor presented with discomfort in the right maxillary sinus area. A computed tomography scan revealed a well‐demarcated unilocular radiolucency within the right maxillary antrum, associated with an endodontically treated maxillary first molar. Surgical excision was performed under general anesthesia via the Caldwell‐Luc approach. The antral wall was reconstructed using titanium mesh and a collagen membrane. The excised specimen was submitted for histopathological evaluation. Histological examination confirmed a diagnosis of intraosseous lipoma, characterized by mature adipocytes in lobules surrounded by woven and cortical bone. No cellular atypia was observed, and surgical margins were clear. Postoperative healing was uneventful.

**Conclusion:**

This case highlights the diagnostic and therapeutic challenges associated with rare maxillary intraosseous lipomas. The wide variability in clinical and radiological presentation makes diagnosis difficult without histopathological confirmation. As the first reported case of a maxillary sinus intraosseous lipoma in an Asian patient, it expands the documented spectrum of this rare entity. Further case documentation and studies are required to better understand the etiology, presentation, and management of this condition.

## 1. Introduction

Pathologies of the maxillary sinus originate from a variety of factors, including inflammatory, allergic, odontogenic, traumatic, cystic, and neoplastic etiologies [1, 2]. Research indicates that inflammatory lesions and cystic conditions are the most common pathologies affecting the maxillary sinus [3]. Clinical manifestations can range from incidental asymptomatic findings to facial pain, nasal obstruction, ear pain, and dental pain [1]. It is well established that there is a connection between dental problems and sinus conditions; for instance, dentigerous cysts linked to ectopic upper third molars have been documented [2, 4]. As with any medical condition, obtaining a comprehensive patient history, conducting a clinical examination, and utilizing radiographic imaging are crucial for forming a differential diagnosis of maxillary sinus disorders. The diagnosis of cystic or neoplastic lesions in the maxilla presents considerable challenges due to a wide range of potential conditions. Surgical excision of these lesions is typically recommended to confirm a histopathological diagnosis and to mitigate potential complications, including nasal obstruction, infection, and malignant transformation [1–4]. Several surgical techniques for the excision of maxillary sinus lesions have been documented, such as the midface degloving approach, the Caldwell‐Luc approach, and endoscopic surgery [5, 6].

Among the various types of maxillary tumors, lipomas, although rare, may occur. Lipomas are benign neoplastic proliferations composed of mature adipocytes. They are the most common mesenchymal neoplasms, typically found on the trunk and limbs [7]. Lipomas in the head and neck regions are less common, accounting for only 15%–20% of all such tumors [8]. Although these lesions often present as long‐standing, soft, nodular, and asymptomatic swellings covered by normal mucosa or skin, intraosseous lipomas have also been reported [9]. Intraosseous lipomas are exceedingly rare, comprising only 0.1% of all bone tumors [10]. Intraosseous lipomas have been reported across various skeletal sites, including the long bones, calcaneus, and cervical spine. Furthermore, these lesions have also been identified in flat bones, with roughly 20% of all documented cases occurring in the head and neck region [11]. A literature search was conducted across three databases (PubMed, EMBASE, and Scopus), using a combination of key terms related to “intraosseous lipoma,” “maxilla,” and “sinus.” Only seven cases of intraosseous maxillary lipoma have been reported in the literature since 1992. Thus far, three cases have been reported in the Asian population since 1992 [12–14]. This is the second reported case of a rare intraosseous lipoma located within the maxillary sinus and the first such case in an Asian individual.

## 2. Case Report

A 41‐year‐old female patient with a history of thalassemia minor was referred to an oral and maxillofacial surgeon by the otolaryngologist for evaluation of a radiolucency that was discovered on a computed tomography (CT) scan of the paranasal sinuses. She first presented with discomfort in the sinus area. Clinical examination was unremarkable, with no signs of expansion or inflammation.

**Figure 1:**
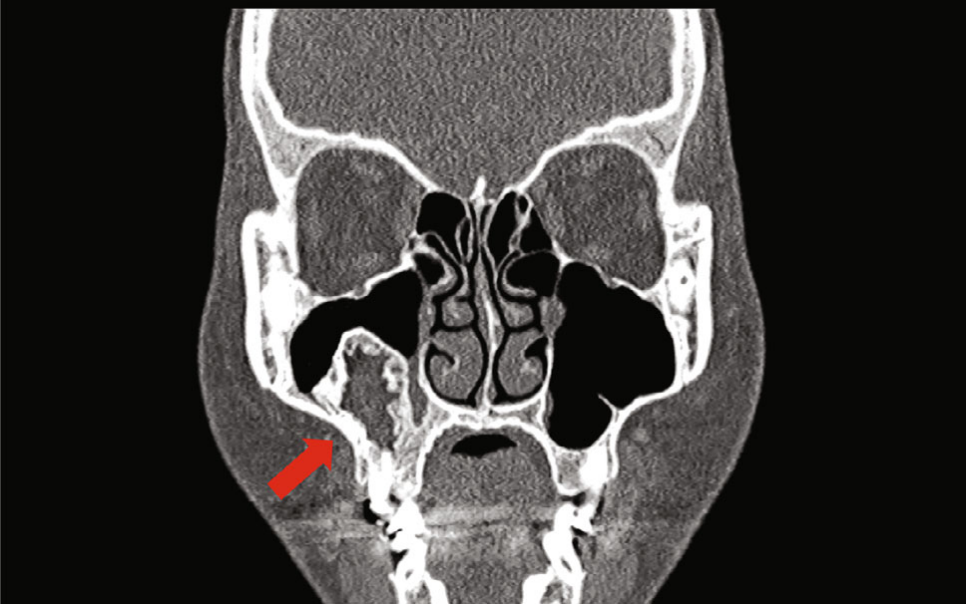
Coronal view of the CT scan reveals a well‐demarcated unilocular radiolucency in the right maxillary antrum.

The contrast‐enhanced CT scan revealed a well‐demarcated unilocular radiolucency with thick, trabeculated cortical margins within the right maxillary antrum (Figure 1). This lesion is associated with the endodontically treated right maxillary first molar and measured approximately 1.9 cm (anteroposteriorly) × 2.3 cm (mediolaterally) × 3.0 cm (superoinferiorly). No aggressive bony or root resorption was observed, and the right maxillary alveolar plate remained intact.

**Figure 2:**
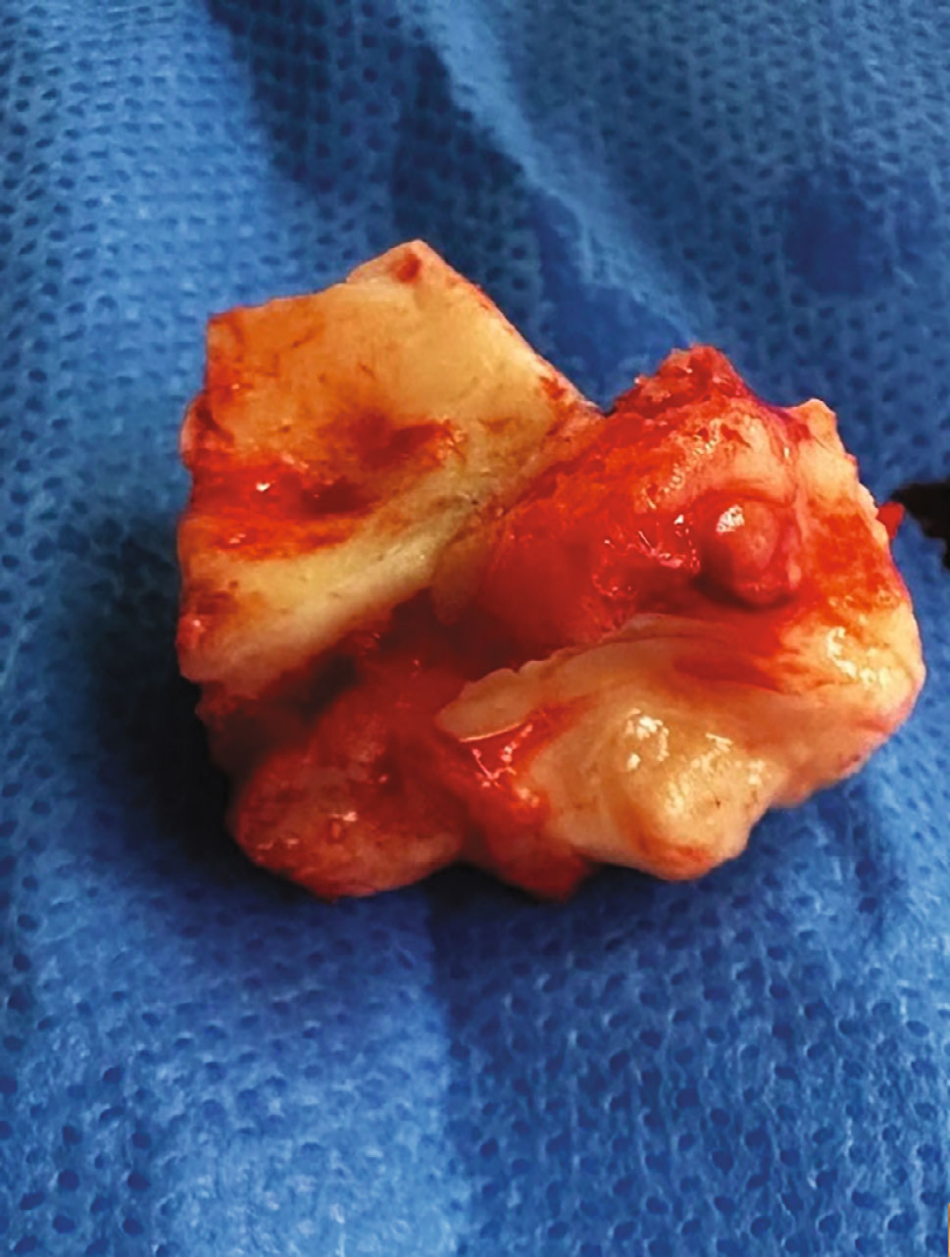
Clinical picture of the excised specimen.

The procedure was performed with the patient under general anesthesia. A mucoperiosteal flap was reflected, and the lesion was excised en bloc using a surgical saw and osteotome via the Caldwell‐Luc approach (Figure 2). Special care was taken during the osteotomy of the alveolar plate. Piezosurgery was employed to avoid any inadvertent damage to the roots of the maxillary teeth. The antral wall was reconstructed with fixed titanium mesh and collagen membrane.

**Figure 3:**
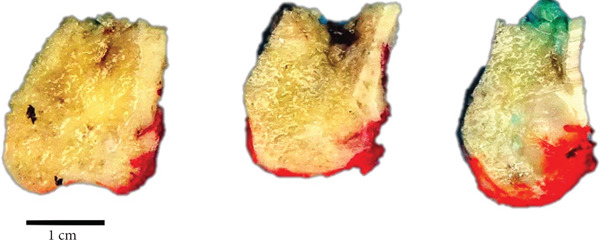
Gross appearance of the intraosseous lipoma.

The gross specimen (Figure 3) comprised a partially mucosa‐lined bony tissue measuring 2.5 cm (superoinferiorly) × 2.5 cm (anteroposteriorly) × 2 cm (mediolaterally). The cut sections showed an ill‐defined, pale, soft lesion. It measured 1.5 cm (superoinferiorly) × 1.2 cm (mediolaterally) × 1 cm (anteroposteriorly).

**Figure 4:**
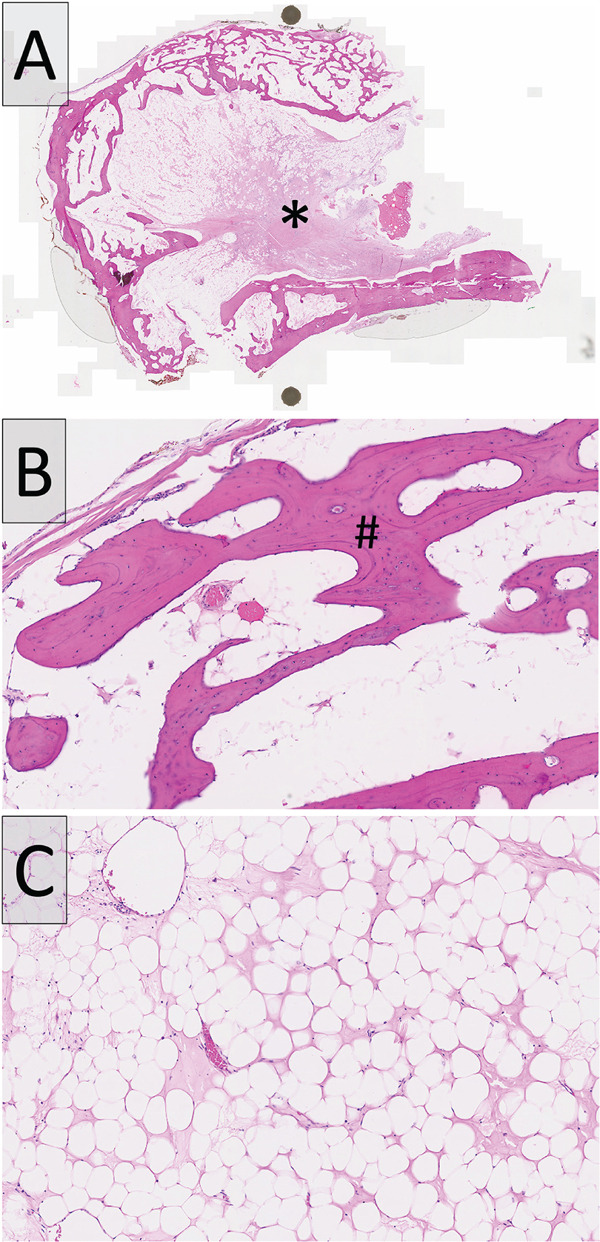
H&E sections showed (A) polypoidal bone‐covered nodule, with the asterisk (∗) denoting the lesion. (B) The lesion is covered by mature bone (#). (C) Sheets of mature adipocytes.

Histopathological examination revealed proliferation of mature adipocytes in lobules bordered by woven bone and rimmed peripherally by the cortical bone (Figure 4). The margins were clear. There were also procedure‐related changes. No significant cellular atypia was observed. A diagnosis of intraosseous lipoma was made.

The patient was evaluated at 1 week and subsequently at 3 months, during which complete wound healing was observed. The patient has remained asymptomatic. Annual clinical and radiographic examinations are planned to ensure ongoing clinical surveillance.

## 3. Discussion

To the best of our knowledge, this is the first report of an intraosseous lipoma occurring within the maxillary sinus in an Asian patient. Intraosseous lipomas may raise diagnostic and management conundrums, and further characterization of this rare lesion is required to optimize patient outcomes.

Intraosseous lipomas are exceptionally rare, particularly in the jaw. Kochaji et al. conducted a literature review and found only 30 reported cases of intraosseous lipoma in the jaw [9]. Our own literature search found only seven cases of maxillary intraosseous lipoma [12–18]. The findings of our review are summarized in Table 1. Owing to the limited number of published cases, significant heterogeneity was observed in the clinical and radiographic presentation of maxillary intraosseous lipomas, reflecting the diverse manifestations of this uncommon entity. Of the reported cases, the patient′s age ranged from 17 to 66 years, with a mean age of 42. Intraosseous lipomas in the maxilla are most commonly reported at the maxillary tuberosity (five of seven cases reported). Moreover, a female predominance was observed (57%).

**Table 1:** Summary of reported cases of maxillary intraosseous lipoma.

**Author**	**Sex**	**Age**	**Ethnicity**	**Location**	**Clinical presentation**	**Radiographic presentation**	**Treatment**
To et al. 1992 [14]	F	66	Chinese	Left maxillary tuberosity	Chronic pain	Expanded maxillary tuberosity with a central radiopacity	Surgical excision
Sakashita et al. 1998 [13]	M	17	Japanese	The apex of the retained left maxillary second deciduous molar	Asymptomatic	Well‐defined unilocular radiolucency	Enucleation
Uysal et al. 2007 [17]	M	15	Turkish	Right face	Disfigurement	Invasive radiopacity that involves the right maxillary sinus, maxilla, and zygomatic bone	Surgical excision
Morais et al. 2011 [18]	F	39	Brazilian	Periapical region of the right maxillary third molar	Discomfort of tooth 18	Apical radiolucency	Lesion found associated with extracted tooth 18
Tabakovic et al. 2018 [16]	F	43	Serbian	Left maxillary tuberosity	Painful bony swelling 2 years after extraction	Well‐defined radiopacity	Enucleation
Babu et al. 2019 [12]	M	52	Indian	Left maxillary tuberosity	Painful bony swelling	Ill‐defined radiolucency	Surgical excision
Queiroz et al. 2023 [15]	F	62	Brazilian	Bilateral maxillary tuberosity	Bilateral maxillary growth that was causing misfit of a provisional partial denture	Mixed radiolucent–radiopaque image with ill‐defined borders on the right side of the maxilla and an ill‐defined radiolucency on the left side	Surgical excision
Current case	F	41	Chinese	Right maxillary antrum	Discomfort in the sinus area	Well‐demarcated unilocular radiolucency with thick, trabeculated cortical margins	Surgical excision

In recent years, three cases of maxillary tuberosity intraosseous lipomas have been reported [12, 15, 16]. In these three cases, the patients were middle‐aged, and all lesions occurred in the maxillary tuberosity, which differs from the location of the maxillary sinus in our case. Radiologically, Tabakovic et al. [16] reported that the case showed a well‐defined radiopacity, which contrasts with our radiolucent presentation. Babu et al. [12] and Queiroz et al. [15] reported that their cases showed ill‐defined appearances, which may raise suspicion of a more sinister lesion. However, Uysal et al. [17] reported an intraosseous lipoma that invaded the maxillary bone. As such, histological diagnosis is usually required in addition to clinical and radiological diagnoses.

The etiology of intraosseous lipomas remains unclear. Several theories have been proposed, including a secondary bone response following trauma, a healing process after osteonecrosis, and the development of a primary benign neoplasm [4, 5]. Similar to the cases reported by Sakashita et al. and Morais et al., the lesion in the present case was associated with a tooth. However, a direct cause‐and‐effect relationship cannot be established.

Intraosseous lipomas are typically asymptomatic and discovered incidentally [9]. Interestingly, our review indicates that pain and swelling are common features of maxillary lesions [12, 14–16, 18]. Disfigurement is a serious but rare complication [17]. Maxillary intraosseous lipomas exhibit a diverse array of radiographic characteristics. These include unilocular radiolucencies, well‐defined radiopacities, mixed radiopacity and radiolucency patterns, and invasive lesions [12–18]. The present case exhibited mild discomfort and a well‐defined unilocular radiolucent lesion characteristic of those found in long bones [19]. Establishing a diagnosis based on clinical and radiographic findings has proven unreliable. Potential differential diagnoses include nonossifying fibroma, fibrous dysplasia, solitary cyst, giant‐cell bone tumor, bone infarct, cartilaginous neoplasms, and osteolipoma [20, 21]. A biopsy is required to make a definitive diagnosis.

Adipocytic lesions that can occur in bones include lipomas, hibernomas, and liposarcomas [10, 22]. The findings in this case were those of a lipoma, which is characterized by lobules of mature adipocytes, without significant atypia. Hibernomas are tumors of brown fat cells, which appear histologically as cells with pale to granular eosinophilic cytoplasm and small central nuclei, findings that are distinctive from lipomas [22]. Liposarcomas, such as well‐differentiated liposarcomas, often show atypical tumor cells with enlarged and hyperchromatic nuclei, which were not seen in this case [23]. Taken together, the clinical, radiological, and histological findings support the diagnosis of intraosseous lipoma.

Osteolipoma is another uncommon lipoma variant reported in the head and neck region. As of March 2024, a recent case series and literature review identified 54 such cases [24]. Clinically and radiographically, osteolipomas may resemble intraosseous lipomas, presenting as asymptomatic, slow‐growing masses with a mixed radiolucent–radiopaque appearance [24, 25]. Histologically, osteolipomas are characterized by mature adipocytes interspersed with foci of trabecular bone without evidence of atypia [21, 24, 25]. In the present case, the lesion originated intraosseously, featuring lobules of mature adipocytes demarcated by the bone, and lacked osseous metaplasia within the adipose tissue, thereby supporting a diagnosis of intraosseous lipoma over osteolipoma.

Various subtypes of intraosseous lipomas have been described. The most common variant is fibrolipoma, distinguished by a substantial fibrous component interspersed among the lobular arrangements of adipose cells [7]. Milgram devised a three‐stage histological classification system for intraosseous lipomas: Stage I, a lesion comprising mature adipose cells without calcification; Stage II, a predominantly fatty lesion with necrosis and focal calcification or ossification; and Stage III, a fat‐containing lesion exhibiting multiple necrotic areas, extensive calcification, and cystic degeneration [26]. The present case aligns with the Stage I classification.

Surgical excision is the treatment of choice for intraosseous lipoma. No recurrence has been reported. Stage III lipomas have been suggested to be at risk of transformation into osteosarcoma; however, this has not been observed in intraosseous lipomas [26]. In the present case, en bloc excision of the lesion was performed, and a clear margin was obtained.

## 4. Conclusion

In conclusion, this case report describes a uniquely rare intraosseous lipoma of the maxillary antrum. To our knowledge, this is the first reported case of intraosseous lipoma within the maxillary sinus in an Asian patient. Our literature review revealed a wide range of clinical and radiographic presentations of maxillary lesions. However, the etiology, characteristics, and risk of malignant transformation remain unclear owing to the scarcity of reported cases. Consequently, further documentation and research are required to better understand this disease.

## Ethics Statement

In all participating institutions, single‐patient case reports that are fully deidentified are exempt from Institutional Review Board (IRB) review. This study complied with the principles outlined in the Declaration of Helsinki. Written informed consent for publication, including the use of images, was obtained from the patient.

## Disclosure

All authors approved the final manuscript.

## Conflicts of Interest

The authors declare no conflicts of interest.

## Author Contributions

**Eng Seng Yeoh:** conceptualization, methodology, investigation, data curation, visualization, writing – original draft, project administration. **Tzy Harn Chua:** formal analysis (histopathology), validation, resources, writing – review and editing. **Jacqueline S. G. Hwang:** formal analysis (histopathology), validation, resources, writing – review and editing. **Sathiyamoorthy Selvarajan:** formal analysis (histopathology), validation, writing – review and editing. **Noah B. T. Teo:** conceptualization, investigation, supervision, writing – review and editing.

## Funding

No funding was received for this research.

## Data Availability

Data available within the article or its supplementary materials.
